# Sex hormone therapy's effect on dry eye syndrome in postmenopausal women: A meta-analysis of randomized controlled trials

**DOI:** 10.1097/MD.0000000000012572

**Published:** 2018-10-05

**Authors:** Chao Liu, Kun Liang, Zhengxuan Jiang, Liming Tao

**Affiliations:** a Department of Ophthalmology, the Second Hospital Affiliated to Anhui Medical University, Hefei, Anhui; b Department of Ophthalmology, the First Affiliated Hospital of Bengbu Medical College, Bengbu PR China.

**Keywords:** dry eye, postmenopausal women, sex hormone

## Abstract

The purpose of the study to assess the efficacy of sex hormone therapy in the treatment of dry eye syndrome in postmenopausal women.

The following electronic databases were searched without language restrictions: PubMed, Embase, Cochrane, and the Chinese Biomedical Database. Two reviewers collected all the literature, which was searched for relevance in English and Chinese from January 1990 to July 2017. Both of the reviewers screened documents independently, identifying the studies that met the inclusion criteria. Then, the included studies were evaluated, and the data were extracted and conversed dependently. Finally, Review Manager 5.3 (offered by the Cochrane collaboration) was used to complete the meta-analysis. An integrated mean difference (MD) with its corresponding 95% confidence interval (CI) was calculated.

A total of 358 patients with dry eye were enrolled in 7 randomized controlled trials (RCTs). We observed statistically significant improvements in the Schirmer's test scores (MD, 2.06; 95% CI, 0.74–4.46; *I*^2^ = 97%; *P* = .006) after sex hormone treatment. However, the scores for tear breakup time (TBUT) (MD, 1.28; 95% CI, −1.03 to 4.68; *I*^2^ = 99%; *P* = .21) and the ocular comfort index (OCI) (MD, −1.12; 95% CI, −4.42 to 1.98; *I*^2^ = 95%; *P* = .48) were not improved.

This meta-analysis of 7 RCTs suggests that sex hormone therapy may be associated with better Schirmer's test scores. However, no significant differences were detected in the TBUT and OCI test scores. Consequently, sex hormone therapy has a potentially useful role in the effective management of postmenopausal women with dry eye syndrome.

## 1 Introduction

Dry eye syndrome is one of the main reasons for patients visiting an ophthalmologist.^[1,2]^ Dry eye is defined as a tear film disorder due to tear damage caused by tear loss or excessive evaporation, and it is associated with ocular discomfort.^[3]^ Dry eye syndrome usually occurs in people over 65 years of age.^[4]^ In the United States, about 3.23 million women are affected by dry eye syndrome.^[5]^ In addition, as several large studies have emphasized, dry eye selectively affects women.^[5,6]^ Dry eye syndrome can cause debilitating symptoms including burning, foreign body sensation, and decreased vision, and affect daily living activities.^[7]^ Currently, there are many approaches to treat dry eye syndrome including patient education, lacrimal substitute, anti-inflammatories, secretagogues, fatty acids, and antioxidants.^[8]^ However, sex hormones might have an impact on the occurrence and development of dry eye syndrome, especially in postmenopausal women.^[9]^

Several previous studies have attempted to demonstrate the efficacy of sex hormones in the treatment of dry eye syndrome.^[10–12]^ However, the results have been conflicting, and there has been no definite conclusion. For example, some randomized controlled trials (RCTs) showed improvements in the tear breakup time (TBUT),^[10,11]^ but others did not.^[12–14]^ Furthermore, these RCTs also measured many different clinical trials (TBUT, Schirmer's test, ocular comfort index [OCI] dryness scores, etc.). This inconsistency may be due to influences of between-study heterogeneity, such as differences in dry eye syndrome severity, diagnostic criteria, and therapeutic methods. In Golebiowski's study,^[12]^ transdermal testosterone and estrogen were used to treat dry eye syndrome. However, in Scuderi's study, phytoestrogen was used to treat postmenopausal women with dry eye syndrome.^[13]^ Hence, the precise roles and advantages of sex hormone therapy in treating dry eye syndrome in postmenopausal women remain unclear and are the subject of debate. In order to provide more authentic and comprehensive evidence for the effectiveness of hormone replacement therapy (HRT), we conducted this meta-analysis of RCTs examining sex hormone therapy in postmenopausal women with dry eye syndrome.

## 2 Materials and methods

The meta-analysis was performed as described in the Cochrane Systematic Assessment Intervention Handbook and the Preferred Reporting Items for Systematic Reviews and Meta-analyses Statement^[15]^; standard systematic review techniques were followed throughout the entire process.

### 2.1 Literature search

For the literature search, the following databases were used without language restrictions (for the period from January 1990 to January 2017): PubMed, Embase, Cochrane, and the Chinese Biomedical Database. The search terms were as follows: “Postmenopausal,” “Dry eye syndrome,” “Keratoconjunctivitis Sicca,” “Xerophthalmia,” “Sex hormone,” and “Sex hormone therapy.” All references in the retrieved articles were scanned to identify other potentially available reports. A total of 280 papers were identified in the initial search, and 7 were included in the final analysis (Table 1). The ethical approval of the present study is not necessary because this is a meta-analysis, which is based on published literature, and no new human participants are involved in this study.

**Table 1:**
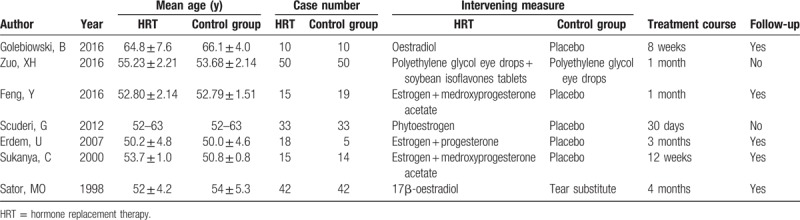
Study characteristics of included studies.

### 2.2 Assessment of risk of bias

We evaluated all the biased risks incorporated into the study using the Cochrane Bias tool.^[14]^ Two authors (Chao Liu and Kun Liang) assessed the bias risk and resolved the differences independently.

Each domain listed in the Cochrane risk of bias tool was evaluated and rated as one of three risk levels: low, unclear, and high. The risk of bias was determined using the standards defined in the Cochrane handbook.

### 2.3 Inclusion and exclusion criteria

Two reviewers independently selected eligible studies. If the 2 judges encountered disagreements, they were resolved through discussion with a third reviewer. The inclusion criteria were as follows:

1. Study design: All randomized controlled studies with complete data regarding the association between dry eye and hormone therapy were considered eligible.
2. Patient type: Patients were postmenopausal women with dry eye, not restricted by race.
3. Interventions: The intervening measures in the experimental group had to include, but were not limited to, sex hormones, while treatments in the control group could not contain sex hormones. Other treatments should be consistent between the 2 groups.
4. The ending index: Report with at least one experimental result (TBUT, Schirmer's test, and OCI score).
5. This study excluded letters, comments, repetitive publications, conference summaries, unqualified control groups, and full text without original data.

### 2.4 Data synthesis

The data were analyzed using Review Manager 5.3 software. The continuum and dichotomous variables were analyzed by mean difference (MD) and risk ratio, respectively. We have used the 95% confidence interval (CI) to calculate all the measures of the effect. The *I*^2^ statistic was used to assess the statistical heterogeneity, and *I*^2^ > 50% and *P* < .1 indicated significant heterogeneity. In these cases, the random effects model was used; otherwise, a fixed effects model was used.^[16]^

In order to evaluate the robustness of the results, a sensitivity analysis was planned, and each study in the meta-analysis was excluded in turn. To investigate the impact of individual studies on the pooled estimates, each study in the meta-analysis was excluded in turn utilizing leave-one-out cross-validation.

## 3 Results

### 3.1 Literature retrieval results

**Figure 1:**
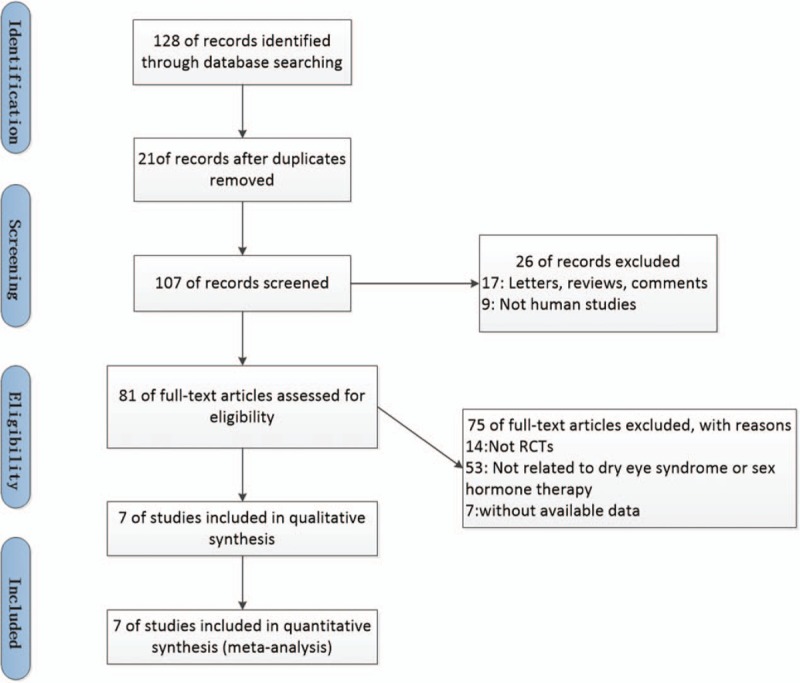
Flow chart showing study selection procedure. This meta-analysis included seven RCT studies.

The search found 128 citations, of which 47 were excluded through a preliminary search and screening of the titles and abstracts. After further consideration of the remaining 81, we excluded 74 studies for the following reasons: 14 not RCTs, 53 not related to dry eye syndrome or sex hormone therapy, and 7 without available data. Finally, the meta-analysis included 7 studies.^[10–14,17,18]^ A detailed description of the search and selection process is shown in Figure 1.

### 3.2 Study characteristics

Seven studies reported on the participants: 185 in the HRT group and 173 in the control group. Two were conducted in China,^[11,14]^ and 1 each in Turkey,^[10]^ Australia,^[12]^ Italy,^[13]^ Austria^[17]^, and Thailand.^[18]^ The main features of the 7 studies are presented in Table 1.

### 3.3 Risk of bias

**Figure 2:**
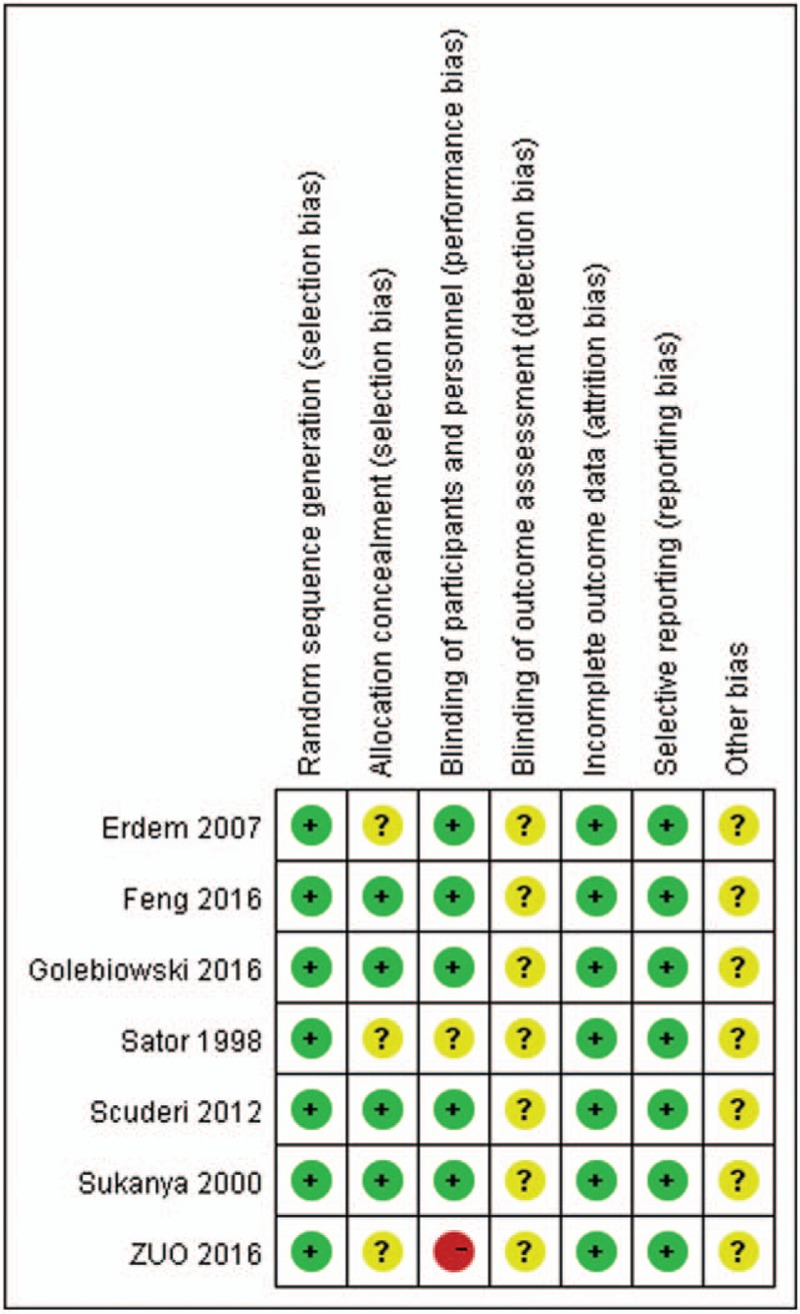
Risk of bias assessment of included studies.

In order to assess the risk of bias, each study was analyzed using the Cochrane Collaboration Organization tool^[19]^ (Fig. 2).

### 3.4 Quantitative analyses

#### 3.4.1 Tear breakup time

**Figure 3:**
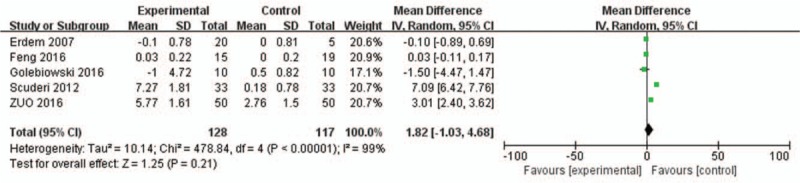
Graph showing the effect of HRT on the scores of TBUT. The squared size of the shadow is proportional to the percentage weight of each study. The horizontal lines stand for 95% CIs. The diamond data flags represent the pooled WMD. The random effect model was applied. WMD = weighted mean difference.

Five of the included trials reported on the TBUT in sex hormone therapy and included control groups. A meta-analysis was performed on five studies of mean standard deviation values, revealing that patients with dry eye syndrome who received the intervention of sex hormone therapy had significantly higher TBUTs than those in control groups (Weighted Mean Difference [WMD] = 1.82, 95%CI = −1.03 to 4.68; *P* = .21) (Fig. 3).

#### 3.4.2 Schirmer's test

**Figure 4:**
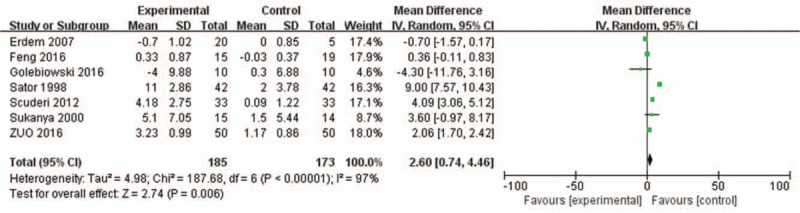
Graph showing the Schirmer's test results affected by sex hormone therapy. The squared size of the shadow is proportional to the percentage weight of each study. The horizontal lines stand for 95% CIs. The diamond data flags represent the pooled WMD. The random effect model was applied. WMD = weighted mean difference.

Seven of the included trials reported on Schirmer's test scores. The meta-analysis showed that patients with dry eye syndrome who were treated with sex hormone therapy had significantly higher Schirmer's test result than those in control groups (WMD = 2.60, 95% CI = 1.70–2.42; *P* = .006) (Fig. 4).

#### 3.4.3 OCI score

**Figure 5:**
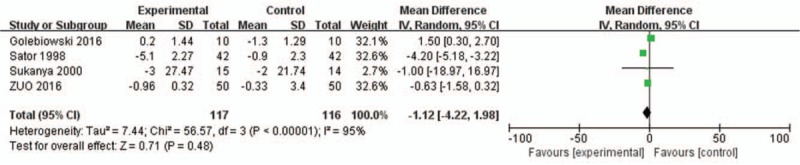
Graph showing the effect of HRT on the scores of OCI dryness. The squared size of the shadow is proportional to the percentage weight of each study. The horizontal lines stand for 95% CIs. The diamond data flags represent the pooled WMD. The random effect model was applied. WMD = weighted mean difference.

Four trials reported on OCI scores. The meta-analysis showed that the dry eye patients treated with sex hormone therapy did not have significantly higher OCI scores (WMD = −1.12, 95% CI = −4.22 to 1.98; *P* = .48) (Fig. 5).

### 3.5 Heterogeneity and sensitivity analysis

Significant heterogeneity was detected in the TBUT test (heterogeneity: *P* < .00001; *I*^2^ = 99%), Schirmer's test (heterogeneity: *P* < .00001; *I*^2^ = 97%), and OCI scores (heterogeneity: *P* < .00001; *I*^2^ = 95%), respectively. Therefore, the one-by-one method was used to exclude the studies to determine the reason for this heterogeneity. However, neither the heterogeneity nor the results changed significantly. After the exclusion of studies with lower methodological quality, the sensitivity analysis revealed no significant changes.

### 3.6 Publication bias

**Figure 6:**
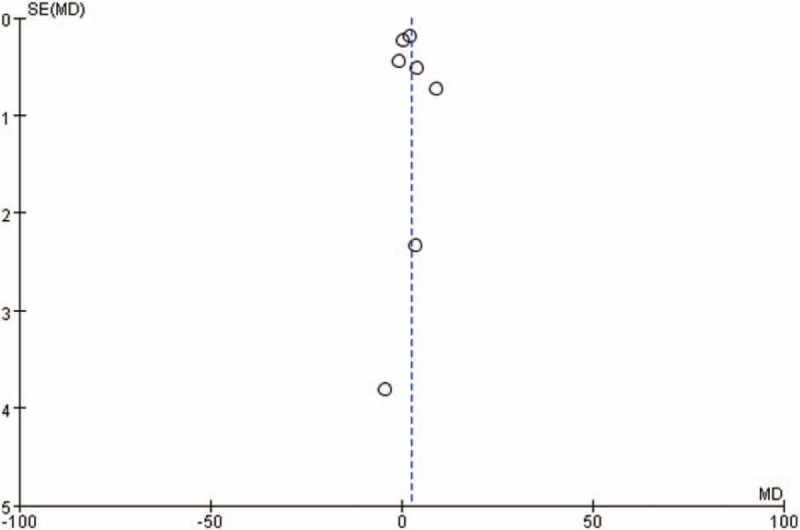
Funnel plot of all the seven studies.

In the present meta-analysis, funnel plots (Fig. 6) of the included studies suggested that publication bias were less likely among the 7 studies.

## 4 Discussion

Dry eye syndrome is defined as a tear film disorder due to tear damage caused by tear loss or excessive evaporation, and it is associated with ocular discomfort.^[1]^ Dry eye syndrome is a common disease in women that frequently manifest following the onset of menopause,^[2]^ especially in postmenopausal women. In women, it has been suggested that sex hormones may help to regulate the function of the tear film and maintain the dynamic balance of the ocular surface as compared to older men.^[17,18,20]^ This notion is also based on the observation that sex hormone receptors can be detected in the ciliary body, iris, retina, meibomian glands, and epithelium of the lens.^[21–25]^

Currently, HRT is commonly used in postmenopausal women to relieve symptoms associated with sex hormone deficiency.^[13]^ However, a study reports that estrogen supplementation may worsen ocular symptoms in postmenopausal women with dry eye, although no impact of testosterone therapy on symptoms was apparent.^[12]^ The clinical efficacy of HRT in dry eye syndrome remains controversial. Therefore, a meta-analysis of RCTs was conducted to provide more authentic and comprehensive evidence for the effectiveness of HRT in postmenopausal women with dry eye syndrome.

In this meta-analysis, the possible efficacy of sex hormone therapy in postmenopausal women with dry eye syndrome has been investigated. This study is the first meta-analysis detecting the effect of sex hormone therapy in the treatment of postmenopausal women with dry eye syndrome. A total of 7 studies were included, and the Cochrane funnel was found to have no publication bias (after the sensitivity analysis, the overall results did not change significantly).

In this study, pooling the results of the available RCTs, we found that HRT improved the Schirmer's test results of postmenopausal women with dry eye syndrome but not the results of the TUBT and OCI. The inclusion criteria and therapeutic effect assessment indexes in the included studies were not exactly the same; we speculated that at least 3 reasons were involved. First, there were significant differences in the interventions used in the experimental group (from estradiol to estrogen + progesterone, even to Chinese medicine, and their concentrations and dosages were also different). Therefore, the effects were very different. For instance, estrogen + progesterone has been proven to have a strong therapeutic effect.^[10,12]^ Second, only 7 studies were available, and the number of patients enrolled was small, which could lead to statistical differences. Third, not all of the research quality was sufficient, and the test reports were not exactly the same. For example, Sator^[17]^ and Sukanya^[18]^ only reported ST and OCI scores.

Therefore, there are some limitations of this meta-analysis, which should be resolved: First, our results were based on unadjusted estimates; more accurate results would come from other confounding factors, such as age, gender, body mass index, and lifestyle. Second, while the heterogeneity is relatively large in this study, the overall results did not change significantly after the sensitivity analysis. However, the studies included in this analysis are insufficient, especially in terms of a subgroup analysis. Thus, publication bias is likely to exist, although no evidence of such bias was obtained from our statistical tests. Third, the RCTs were from different countries and different races, and were performed in different environments. Therefore, the patients with dry eye syndrome analyzed here may have different sensitivities and responses to the same sex hormone dosages and concentrations. All of these factors may have affected the final results.

Use of topical estradiol is still under investigation and there is an ongoing phase study about such treatment. Systematic and topical use of estradiol also have some side effects, such as nausea, headache, edema, obesity, altered libido, increased cancer risk, etc.^[26,27]^ It should be cautious for dry eye patients with infection when use estradiol for curing the dry eye. With the continuous development of new drugs, we believe that hormone therapy may also be gradually replaced.

## 5 Conclusion

This meta-analysis of 7 RCTs suggests that sex hormone therapy seems to be related to better Schirmer's test results. However, no significant differences were detected in the TUBT and OCI scores. Consequently, sex hormone therapy plays a potentially useful role in postmenopausal women with dry eye syndrome. Therefore, additional larger, well-designed, multicenter RCTs with wider follow-up are needed to improve the credibility of our results.

## Author contributions

**Data curation:** Chao Liu and Kun Liang.

**Formal analysis:** Liming Tao, Chao Liu, Kun Liang, and Zhengxuan Jiang.

**Methodology:** Chao Liu and Kun Liang.

**Software:** Chao Liu.

**Writing – original draft:** Chao Liu.

**Writing – review and editing:** Liming Tao, Chao Liu, Kun Liang, and Zhengxuan Jiang.
